# Diagnostic validity of p16, E-cadherin, cyclin D1, p53, and HPV E6/E7 mRNA in CIN 3-like squamous cell carcinoma of the cervix

**DOI:** 10.3389/fonc.2024.1354838

**Published:** 2024-01-26

**Authors:** Zhao Xing, Shen Danhua, Zhang Xiaobo

**Affiliations:** ^1^ Department of Pathology, The Affiliated Hospital of Chengde Medical College, Chengde, China; ^2^ Department of Pathology, Peking University People’s Hospital, Beijing, China

**Keywords:** CIN 3, CIN 3-like squamous cell carcinoma, cervix, HPV E6/E7, p16

## Abstract

**Objective:**

Cervical intraepithelial neoplasia grade 3 (CIN 3)-like SCC is a recently identified deceptive growth pattern that closely mimics endocervical crypt involvement by CIN 3. As CIN 3-like SCC is indistinguishable from endocervical crypt involvement by CIN 3, it poses a significant challenge for pathologists.

**Method:**

We examined 23 cases of CIN 3-like SCC, 6 of which also had concomitant conventional invasive SCC, and 9 cases of endocervical crypt involvement by CIN 3 as a control group. Immunohistochemistry was used to investigate the expression of p16, E-cadherin, cyclin D1, and p53, and the expression of human papillomavirus (HPV) E6/E7 mRNA, the key virus carcinogen of HPV, was detected. The clinicopathological, immunohistochemical, and molecular characteristics of endocervical crypt involvement by CIN 3, CIN 3-like SCC, and the concomitant conventional invasive SCC element were examined.

**Result:**

CIN 3-like SCC exhibited a characteristic morphology similar to endocervical crypt involvement by CIN 3, with pushing borders invading into the wall of the cervix, often to a significant depth in most cases. Immunophenotypic features of E-cadherin, p16, cyclin D1, and p53 differed between CIN 3-like SCC and conventional invasive SCC, both in staining intensity and region. E6/E7 mRNA expression was higher in CIN 3-like SCC than in endocervical crypt involvement by CIN 3 (P < 0.05).

**Conclusion:**

CIN 3-like SCC is the type of cancer, presenting numerous challenges and potential for confusion as it mimics the phenotypes of endocervical crypt involvement by CIN 3.

## Introduction

Cervical cancer is a prevalent malignancy that poses a significant threat to women’s health. Squamous cell carcinoma (SCC) is the most commonly observed histological subtype, and the various patterns of SCC have notable implications for the prognosis and treatment of cervical cancer. It is crucial to differentiate between infiltrating and preinfiltrating lesions. Typically, the tumor cells of cervical SCC infiltrate as budding, single cells, or small solid nests, displaying characteristic intervening desmoplastic or inflammatory stroma. However, in some cases, invasion becomes challenging to confirm or exclude based on biopsy, conization, or even hysterectomy, as the invasive squamous epithelium exhibits features similar to those of endocervical crypt involvement by CIN 3. Herein, we report on 23 patients with CIN 3-like SCC admitted to Peking University People’s Hospital. We analyzed the infiltration characteristics, immunophenotype, molecular features, and biological behavior to facilitate a more accurate diagnosis of CIN 3-like invasive SCC.

## Materials and methods

### Samples

A total of 23 cases of cervical CIN 3-like SCC and 9 cases of endocervical crypt involvement by CIN 3 were collected from Peking University People’s Hospital from January 2022 to September 2023. The surgical approach included preoperative biopsy, loop electrosurgical excision procedure, conization of the cervix, and radical hysterectomy. All case slides underwent further review and confirmation by two gynecological pathologists. Clinicopathological information, including age, cervical screening cytology, clinical history, colposcopic findings, and tumor stage, was collected. Follow-up information was obtained by tracing the regular reviews of the patients.

### Histological examination

Cervical tissue specimens were fixed with formalin, embedded in paraffin, and stained with hematoxylin and eosin before examination via light microscopy. The diagnosis was strictly confirmed based on the criteria of CIN 3-like SCC as described by Al-Nafussi and Monaghan (1). At low magnification, large and well-circumscribed cellular aggregates were observed, displaying circumscribed pushing borders and characterized by deep invasion into the cervical wall. Contrary to the conventional infiltration pattern, CIN 3-like SCC is more commonly associated with a lymphoplasmacytic inflammatory infiltrate and mild stromal desmoplasia. At high magnification, parakeratotic or necrotic material is often evident in the central cavities of the tumor nests, mimicking endocervical crypt involvement by CIN 3.

### Immunohistochemistry

Immunohistochemical staining was conducted on the paraffin-embedded sections using an automated immunohistochemical staining device. Antibodies against p16, E-cadherin, cyclin D1, and p53 were used for immunohistochemical staining. Positive controls consisted of known positive sections (cervical cancer tissue sections), whereas a negative control was established by replacing the primary antibody with phosphate-buffered saline. Cyclin D1 and p53 staining was localized in the nucleus, p16 staining was observed in the nucleus and cytoplasm, and E-cadherin staining occurred in the cytoplasm and cell membrane.

### Human papillomavirus (HPV) E6/E7 mRNA detection

HPV E6/E7 mRNA detection was performed to identify the key viral carcinogen of HPV in paraffin-embedded cervical tissues (Zhengzhou Kodia Biotechnology Co., Ltd.). All specimens were routinely serially sectioned to 3–5 µm. The lysis solution was added for target RNA in 96 plates, involving hybridization of the target probe with target RNA, signal amplification, and detection using a chemiluminescent fluorescence detector. The amount of target RNA was calculated based on the RNA copy number according to the manufacturer’s instructions. The positive criterion was an RNA copy number of >1.

## Results

The age range of patients with CIN 3-like SCC was 27–71 years, with median and mean ages of 49 and 48 years, respectively. Of the 23 patients with CIN 3-like SCC, 11 underwent total hysterectomy, while 12 underwent cervical conization. Additionally, 21 patients underwent cervical screening cytology, of whom 11 exhibited high-grade squamous intraepithelial lesion (HSIL), 4 low-grade squamous intraepithelial lesion (LSIL), 4 atypical squamous cells of undetermined significance (ASC-US), 1 ASC–cannot exclude HSIL (ASC-H), and 1 negative for intraepithelial lesion or malignancy. Furthermore, of 9 patients with endocervical crypt involvement by CIN 3, 7 underwent cervical screening cytology; 2 exhibited HSIL, 1 LSIL, 3 ASC-US, and 1 ASC-H. Colposcopic findings were available for all patients, and all were confirmed to have cervical lesions. Of the 23 cases of CIN 3-like SCC, only 4 were confirmed as SCC in preoperative biopsy, 3 cases showed suspicious infiltration, and 16 cases were classified as HSIL. After review, the accurate preoperative diagnosis rate was 30.43%. Moreover, of the 23 cases of CIN 3-like SCC, 6 exhibited co-occurrence with conventional invasive SCC, all cases exhibited co-occurrence with endocervical crypt involvement by CIN 3, and 17 cases were classified as stage IA and 6 as stage IB. All patients survived without disease recurrence or metastasis until September 2023.

### Histological features in patients with CIN 3-like SCC

In 17 of the 23 cases of CIN 3-like SCC, the CIN 3-like growth pattern, as a single infiltration pattern, was observed in the background of endocervical crypt involvement by CIN 3, without conventional invasive SCC.

In the 6 cases exhibiting co-occurrence with conventional invasive SCC, the proportion of the CIN 3-like growth pattern varied from 10% to 40%, showing an infiltration pattern predominantly of conventional invasive SCC.

**Figure 1:**
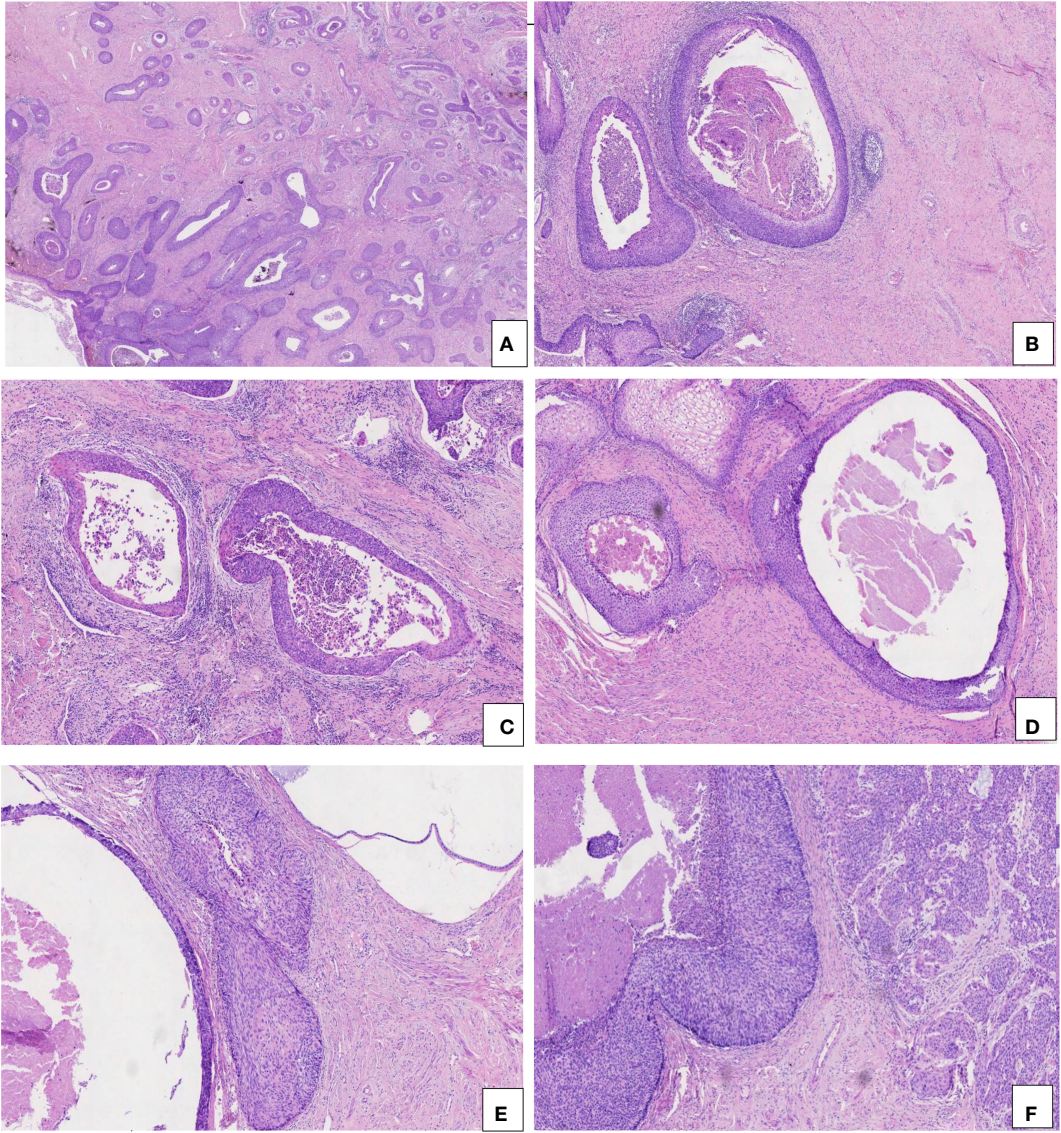
Pathological characteristics of CIN 3-like SCC. **(A)** Well-circumscribed and large cellular aggregates, invading into the wall of the cervix (HE, 2×). **(B, C)** Mild stromal and inflammatory reaction (HE, 5× and 10×). **(D)** Necrosis and dyskeratosis in cells at the center of the larger tumor nests (HE, 10×). **(E)** CIN 3-like SCC (left) co-occurring with endocervical crypt involvement by CIN 3 (right) (HE, 10×). **(F)** CIN 3-like SCC (left) co-occurring with conventional invasive SCC (right) (HE, 1×).

All the CIN 3-like SCC cases showed characteristic morphology, being well-circumscribed and having large cellular aggregates. This appearance is similar to endocervical crypt involvement by CIN 3, with pushing borders invading into the cervix wall, deeply in most cases (**Figure 1A**).

The invasion depth of CIN 3-like SCC ranged from 1.8 to 9.7 mm, with median and mean depths of 9.7 and 7.17 mm, respectively. In the 23 cases of CIN 3-like SCC, the diameter of the large cellular aggregates ranged from 0.8 to 4.6 mm, with median and mean diameters of 2.3 and 2.03 mm, respectively. Unlike conventional epithelial tumors, the stromal reaction in CIN 3-like SCC was often mild and sometimes accompanied by inflammatory cell infiltration (**Figures 1B, C**). Necrosis and dyskeratosis cells could be observed at the center of the larger tumor nests (**Figure 1D**). Among the 23 cases of CIN 3-like SCC, dyskeratosis cells were detected in 12 cases (52.17%), necrosis in 7 cases (30.43%), and both in 4 cases (17.39%). As expected, owing to a lack of sufficient understanding of the special invasion pattern, making an accurate diagnosis, particularly in preoperative biopsy tissue, was challenging. Most cases (17 cases) showed co-occurrence with endocervical crypt involvement by CIN 3 (**Figure 1E**), whereas 6 cases exhibited conventional invasive SCC (**Figure 1F**).

### Immunohistochemistry

**Figure 2:**
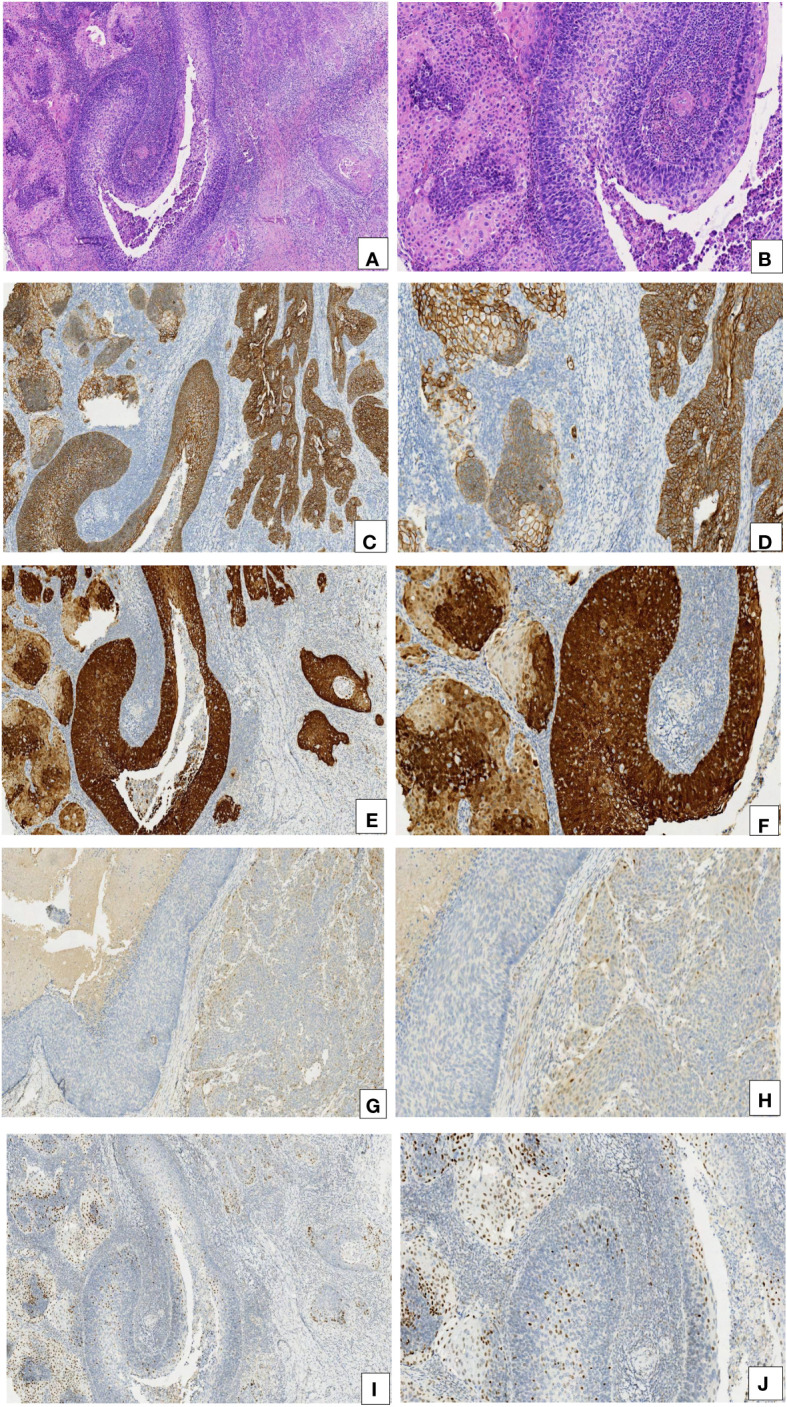
Comparison of the immunohistochemical characteristics of CIN 3-like SCC and the conventional invasive component. **(A, B)** CIN 3-like SCC co-occurring with the conventional invasive SCC (HE, 10× and 20×). **(C, D)** E-cadherin expression was higher in CIN 3-like SCC than in the conventional invasive SCC (IHC, 10× and 20×). **(E, F)** p16 expression was higher in CIN 3-like SCC than in conventional invasive SCC (IHC, 10× and 20×). **(G, H)** Cyclin D1 expression was lower in CIN 3-like SCC than in conventional invasive SCC (IHC, 10× and 20×). **(I, J)** p53 expression was restricted to cells at the center of the cell cluster, whereas it was diffuse in the conventional invasive SCC.

**Figure 3:**
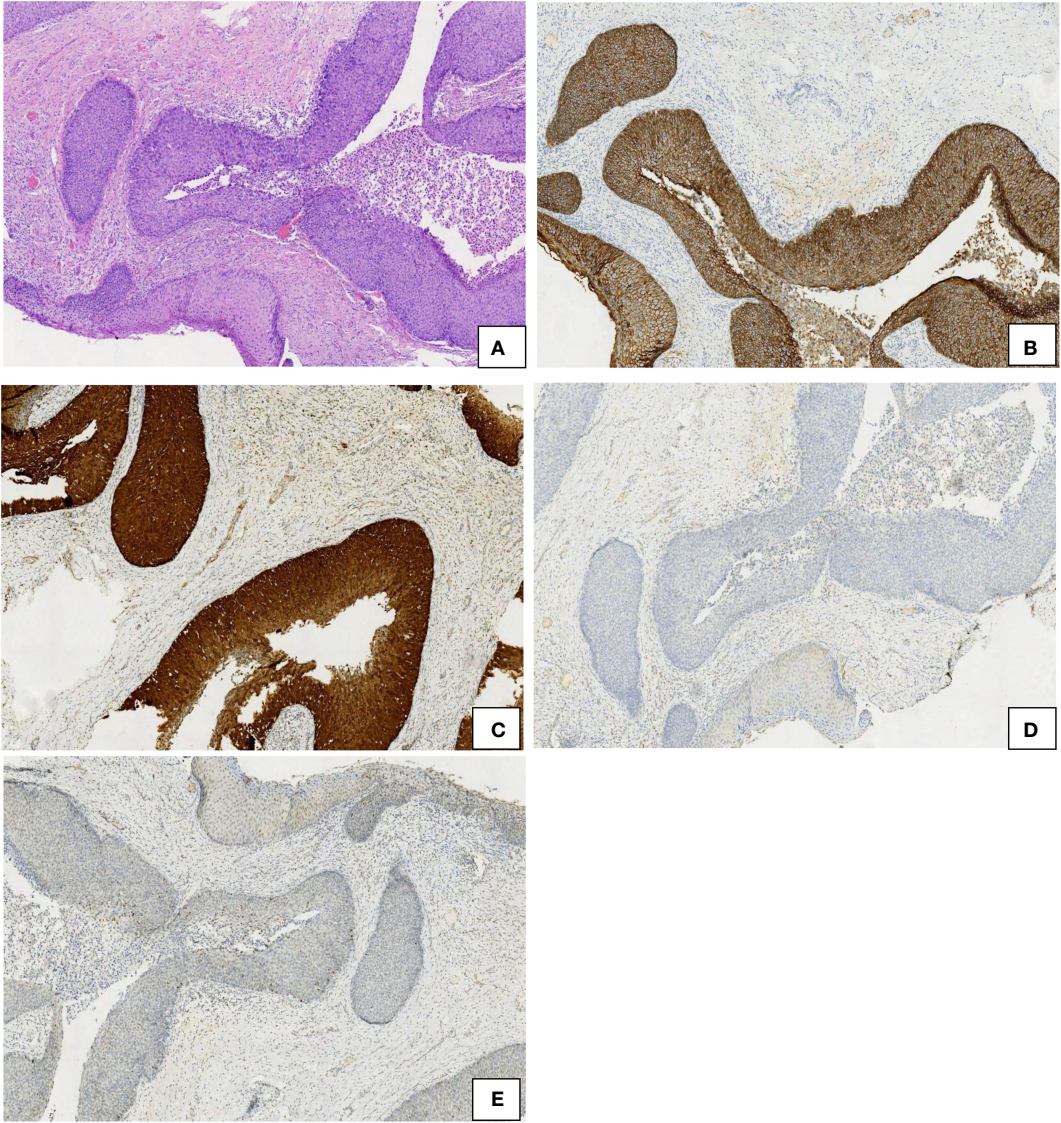
Comparison of the immunohistochemical characteristics of endocervical crypt involvement by CIN 3 (left) and CIN 3-like SCC (right). **(A)** HE, 10×. **(B)** E-cadherin, 10×. **(C)** p16, 10×. **(D)** cyclin D1, 10×. **(E)** p53, 10×.

The expression of E-cadherin, p16, cyclin D1, and p53 was detected via immunohistochemistry. The expression of E-cadherin was found to be higher in CIN 3-like SCC than in conventional invasive SCC. E-cadherin was observed to be diffused in membrane in CIN 3-like SCC, with uneven staining in the infiltrative cells (reduced in some parts). p16 expression was higher in CIN 3-like SCC than in conventional invasive SCC, with uneven staining in the infiltrative cells, reduced in the periphery (stromal interface) of the infiltrative component. Cyclin D1 expression was negative in CIN 3-like SCC, restricted to cells at the periphery (stromal interface) of the infiltrative component. p53 expression was restricted to cells at the center of the cell cluster of tumor cells, while it was diffuse in conventional invasive SCC (**Figure 2**). In contrast, endocervical crypt involvement by CIN 3 showed the same immunophenotypic feature as CIN 3-like SCC (**Figure 3**).

### Comparison of the expression levels of HPV E6/E7 mRNA

**Figure 4:**
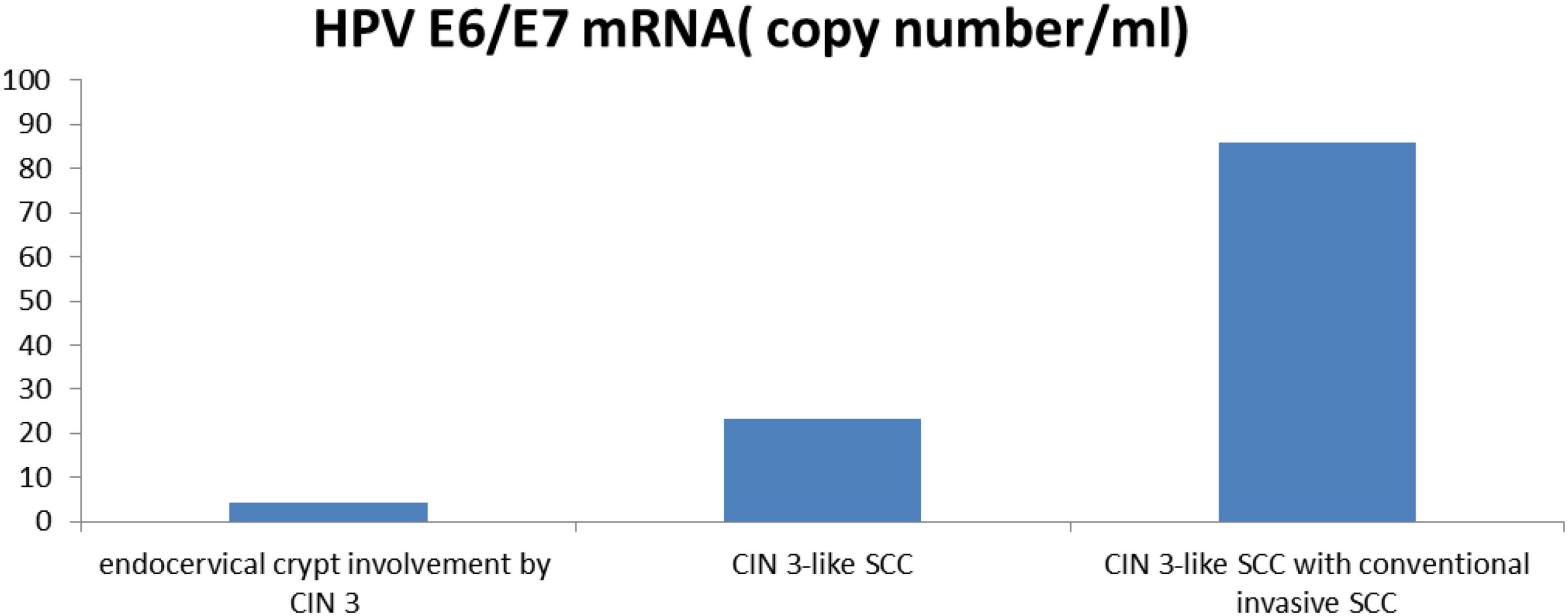
Comparison of the expression levels of HPV E6/E7 mRNA in endocervical crypt involvement by CIN 3, CIN 3-like SCC, and CIN 3-like SCC with conventional invasive SCC.

The expression level of HPV E6/E7 mRNA in 9 cases of endocervical crypt involvement by CIN 3 was 4.43 ± 4.88 (range, 0.989–16.52). In 17 cases of CIN 3-like SCC, the expression level of HPV E6/E7 mRNA was 23.23 ± 24.14 (range, 2.272–84.835). In 6 cases of CIN 3-like SCC with conventional invasive SCC, the expression level of HPV E6/E7 mRNA was 85.88 ± 99.32 (range, 4.775–256.34). Thus, overall, E6/E7 mRNA expression was found to be significantly higher (P < 0.05) in CIN 3-like SCC, with or without the conventional invasive element, than in endocervical crypt involvement by CIN 3 (**Figure 4**).

## Discussion

The World Health Organization is focused on reducing the mortality of cervical cancer through cytological and HPV DNA testing (2). Recently, adolescent girls have been encouraged to receive the HPV vaccine to prevent infection by certain types of the virus, such as HPV16 and HPV18. Early diagnosis and treatment are vital for the prognosis of cervical cancer. For pathologists, distinguishing precancerous lesions from invasive cancer is crucial. Cervical precancerous lesions can be classified into three grades, CIN 1, CIN 2, and CIN 3, representing a series of related events in cervical cancer. Differentiating between HSIL and LSIL is critical for managing and treating cervical squamous lesions. However, it is even more crucial to determine the possible existence of SCC. Accurate pathological diagnosis is related to the stage and significantly influenced by the surgical protocol, especially for young women who wish to preserve their fertility. Generally, pathologists can recognize early infiltration by budding; however, in some cases, differential diagnosis between CIN 3 and early infiltrating cancer can be challenging, particularly in cases with extensive and deep cervical intraepithelial neoplasia glandular involvement. CIN 3-like SCC is the type of cancer mentioned above, presenting numerous challenges and potential for confusion as it mimics the phenotypes of endocervical crypt involvement by CIN 3 (3). Our study aimed to identify the rare pattern of CIN 3-like SCC and analyze the potential role of p16, E-cadherin, cyclin D1, and p53 in its diagnosis.

CIN 3-like SCC is a rare variant, often erroneously diagnosed as endocervical crypt involvement by CIN 3, especially in small biopsy tissues. Such misdiagnosis may even lead to delayed diagnosis and surgery. The most notable feature of CIN 3-like SCC is the presence of circumscribed, large cells with necrosis and dyskeratosis in the center of the cell nests, closely resembling the appearance of executive and deep endocervical crypt involvement. Owing to a lack of knowledge and attention to this unique infiltration method, underestimation of tumor extent and depth of invasion becomes inevitable. When encountering suspected clinical cancer cases, integration with other clinical examinations is essential. The biological behavior is detailed in some reports (4, 5). Retroperitoneal nodes and psoas muscle metastasis of CIN 3-like SCC have been described, exhibiting the same appearance as the primary focus. However, in some cases, the metastatic deposit showed no specific architectural pattern.

The results of the histological diagnosis of CIN 3-like SCC are substantially influenced by the subjectivity of the observer. Therefore, identification of relevant auxiliary indicators may provide a new basis for diagnosis and facilitate early treatment and lesion diagnosis. There is limited information available about the theory behind the CIN 3-like variant. Epithelial–mesenchymal transition (EMT) (6–8) plays a significant role in tumor invasion and metastasis, with associated features including the high expression of cyclin D1 and loss of expression of E-cadherin. Previous studies have suggested that decreased expression of E-cadherin promotes poor adhesion and contributes to invasiveness and a poor prognosis (9–11). Because of the growth pattern characterized by a cell mass with a clear boundary, it is expected that CIN 3-like SCC exhibits an invasion or migration pattern of collective cells. Unlike conventional invasive SCC, the tumor invades as large cohesive aggregates without an obvious stromal reaction, with positive expression of E-cadherin, which mediates the integrity of cell groups. The E-cadherin switch results in the alteration of adherens junctions even in the early phase of cervical squamous, playing a role in cell function, cell adhesion, growth regulation, invasion, and metastasis. In this study, we found that E-cadherin expression was higher in CIN 3-like SCC than in conventional invasive SCC. Endocervical crypt involvement by CIN 3 showed the same immunophenotypic feature as CIN 3-like SCC. Thus, our results indicate that at least E-cadherin could be a key biomarker of CIN 3-like SCC, distinguishing it from conventional invasive SCC, as reported previously (12). We also observed the downregulation of cyclin D1 in CIN 3-like SCC compared to that in the conventional invasive component. As mentioned above, cyclin D1 plays an important role in EMT (13, 14), and our findings confirm such a role in cervical SCC, as indicated by a higher expression in the conventional infiltrative growth pattern. Both E-cadherin and cyclin D1 were found to have no effect on the differential diagnosis between CIN 3-like SCC and endocervical crypt involvement by CIN 3, and this point of view has not been reported in previous studies.

HPV DNA tests detect the presence of the virus by identifying viral DNA, and the results indicate whether there is a viral infection. Conversely, E6/E7 mRNA detection aims to identify key viral carcinogens. The results are used to assess cancer risks; for example, HPV mRNA detection can help detect HPV infections leading to cellular transformation and is actionable. Research on the HPV status of CIN 3-like SCC is extremely limited. Most cases of CIN and conventional SCC are associated with HPV (15, 16). In this study, we focused on HPV status using HPV E6/E7 mRNA detection. Our results indicated that the expression of E6/E7 mRNA was higher in CIN 3-like SCC and conventional infiltrative SCC than in endocervical crypt involvement by CIN 3. These results suggest that the detection of the HPV mRNA may be a promising predictive method for the differential diagnosis between CIN 3-like SCC and endocervical crypt involvement by CIN 3.

Theoretically, for the diagnosis of CIN 3-like SCC, morphology is most important. Based on morphology, the predictive value of immunohistochemistry and molecular detection may be more accurate.

## Data availability statement

The original contributions presented in the study are included in the article/supplementary material. Further inquiries can be directed to the corresponding author.

## Ethics statement

The study was approved by the institutional review board (CWO) of Affiliated Hospital of CHENGDE Medical College. All patients provided written informed consent.

## Author contributions

ZX: Writing – original draft. SD: Writing – review & editing. ZXB: Investigation, Writing – review & editing.
